# Obstetrical outcome valuations by patients, professionals, and laypersons: differences within and between groups using three valuation methods

**DOI:** 10.1186/1471-2393-11-93

**Published:** 2011-11-12

**Authors:** Denise Bijlenga, Erwin Birnie, Ben WJ Mol, Gouke J Bonsel

**Affiliations:** 1 Dept. of Social Medicine, Academic Medical Centre - University of Amsterdam, PO Box 22660, 1100 DD Amsterdam, The Netherlands; 2 Institute of Health Policy and Management, Erasmus MC, PO Box 1738, 3000 DR Rotterdam, The Netherlands; 3 Dept. of Obstetrics and Gynecology, Academic Medical Centre - University of Amsterdam, PO Box 22660, 1100 DD Amsterdam, The Netherlands; 4 Institute of Health Policy and Management, Erasmus MC, PO Box 1738, 3000 DR Rotterdam, The Netherlands

**Keywords:** health outcome valuation, preference, vignettes, psychometrics, pregnancy, obstetrics

## Abstract

**Background:**

Decision-making can be based on treatment preferences of the patient, the doctor, or by guidelines based on lay people's preferences. We compared valuations assigned by three groups: patients, obstetrical care professionals, and laypersons, for health states involving both mother and (unborn) child. Our aim was to compare the valuations of different groups using different valuation methods and complex obstetric health outcome vignettes that involve both maternal and neonatal outcomes.

**Methods:**

Patients (n = 24), professionals (n = 30), and laypersons (n = 27) valued the vignettes using three valuation methods: visual analogue scale (VAS), time trade-off (TTO), and discrete choice experimentation (DCE). Each vignette covered five health attributes: maternal health ante partum, time between diagnosis and delivery, process of delivery, maternal outcome, and neonatal outcome. We used feasibility questionnaires, Generalization theory, test-retest reliability and within-group reliability to compare the valuation patterns between groups and methods. We assessed relative weights from each valuation method to test for consistency across groups.

**Results:**

Test-retest reliability was equal across groups, but different across methods: highest for VAS (ICC = 0.61-0.73), intermediate for TTO (ICC = 0.24-0.74) and lowest for DCE (kappa = 0.15-0.37). Within-group reliability was highest in all groups with VAS (ICC = 0.70-0.73), intermediate with DCE (kappa = 0.56-0.76) and lowest with TTO (ICC = 0.20-0.66). Effects of groups were smaller than effects of methods. Differences between groups were largest for severe health states.

**Conclusion:**

Based on our results, decision making among laypersons should use TTO or DCE; patients should use VAS or TTO.

## Background

In the last decades, new methods on outcome measurement in clinical studies have emerged, particularly in the context of economic evaluation. Instead of conventional primary clinical endpoints specific to the disease, these new methods rely on a generic composite measure, predominantly the quality-adjusted life year (QALY) [1]. However, a major disadvantage of the QALY is that many health outcomes cannot be easily framed into the QALY format, particularly if treatment burden is relevant or if 'outcome' refers to more persons than just the treated patient.

Alternatives to the QALY in such cases are the recently introduced preference- or attitude-based measures. These measures provide a numerical value to a combination of health characteristics specific to the decision problem. Such preference measures, however, explicitly rely on preference statements of 'judges' which may be recruited from various groups of stakeholders, e.g. policy makers, care providers, families, and patients [2]. Generally, heterogeneous responses may occur within and across various groups of stakeholders [3-15]. Part of this heterogeneity may reflect the disagreement between the groups, but another part is more likely caused by method effects [5,16-19]. It is also conceivable that different groups use valuation methods differently, which causes an interaction effect between group and method. We therefore investigate whose values we should use when the health outcomes are complex, using three widely-used valuation methods.

In obstetrics, decision problems involve the health of at least two patients (mother and child). The outcome of both depends on the same treatment. A beneficial treatment for the mother may harm the child and vice versa. Also process outcomes may be relevant, e.g. the mode of delivery. However, the informed decision-making is hampered by a lack of insight to the patient's and the professional's preferences. Also laypersons' preferences have to be taken into consideration in the context of societal decision-making.

In this study we compare the valuations and preferences of three different groups relevant to obstetric decision-making using three different valuation methods. The different groups were young mothers who recently experienced a complicated pregnancy ('patients'), obstetrical care professionals, and laypersons. We use the three often used preference/attitude based methods: The visual analogue scale (VAS), the time trade-off (TTO), and the discrete choice experimentation (DCE). We present typical scenarios that were drawn from a mild risk situation which is relevant in at least 5% of pregnancies. The decision at hand is the one between induction of labour and expectant management in pregnancies complicated by gestational hypertension (GH), pre-eclampsia (PE) and/or intra-uterine growth retardation (IUGR) after 36 weeks of gestation [20-23].

We aim to compare the valuations of different groups using different valuation methods and complex obstetric health outcome vignettes that involve both maternal and neonatal outcomes in order to establish which group, or perhaps group-method combination, should be used for decision making. We will measure feasibility, reliability, and comparability for each combination of group and valuation method. Our hypothesis is that within each group, participants' valuations will show consensus regardless of the valuation method, but that there will be differences in valuations between the three groups. However, we expect that rankings of the health states, as opposed to absolute valuations, will be equal between groups, regardless of valuation method.

## Methods

### Vignettes

First, we performed in-depth interviews with ten patients with GH, PE and/or IUGR and with ten obstetrical care professionals about the physical, psychological, and social burden and other consequences of GH, PE and IUGR. From these qualitative interviews, a list of 42 aspects emerged, which was aggregated into five attributes [24]: 'maternal health ante partum', 'time between diagnosis and delivery', 'process of delivery', 'maternal outcome', and 'neonatal outcome'. The attribute levels were chosen according to interviewees' responses, literature review, and primary and secondary outcome measures from the HYPITAT (ISRCT08132825) [21,22] and DIGITAT (ISRCT10363217) [20,25] trials. Each attribute had 2 to 7 levels; all were defined to be present with certainty (i.e. no risks involved).

We converted the attributes and levels into vignettes containing both a visual and a written representation (Additional file 1). The visual part depicts a time line to visualize the course of maternal and neonatal health over time. The time lines start when GH, PE or IUGR is diagnosed and they end one year post partum. A text box over the maternal timeline depicts the process of delivery: induction of labour, onset of delivery, and mode of delivery. Colours were used to display severity of health states; explanation of the colours and obstetric/perinatal terms were given on a detailed reference sheet (Additional file 2). Details of this procedure are explained elsewhere [24].

### Design

The total number of usable unique vignette pairs was 37,990. Because of this large number of usable vignette pairs we applied an incomplete factorial design of 240 single vignettes for the VAS and TTO, and 120 paired vignettes for the DCE method (for details, we refer to [24]). We checked for the assumptions of orthogonality and level balance.

The 240 (120 paired) vignettes were distributed over six booklets. Each booklet consisted of two parts: 20 panel session vignettes (18 single VAS/TTO vignettes (9 paired DCE vignettes), plus the best and worst possible vignettes which we used for anchoring, and 26 home-assignment vignettes (22 single vignettes (11 paired vignettes), plus 4 single re-test vignettes (2 paired vignettes)). In this study we compared the outcomes between groups using just one of the six booklets. The other five were used for the larger study in which the outcomes of the total design was the objective [24]. All participants of current study valued the same set of 46 vignettes.

### Participants

Participants in the valuation study were 24 patients, 30 obstetrical care professionals and 27 laypersons. The group of patients consisted of women who had a pregnancy that was complicated either by GH, PE, or IUGR and have participated to either the DIGITAT or the HYPITAT trials [22,26]. These women participated in the study within six months after childbirth. The group of obstetrical care professionals consisted of gynaecologists, midwives, and residents in gynaecology, none of them with specific expertise in health state valuation, but all involved in the Dutch Obstetric Consortium (for more information, see http://www.studies-obsgyn.nl). They were recruited by email invitation. The laypersons were men and women over 18 years of age who had previously participated in valuation studies [27]. The laypersons and patients received a €50 participation fee.

### Valuation methods

Each participant valued the single vignettes with a VAS and TTO, and each paired vignette with a DCE.

The VAS is a psychometric rating method with equal-interval categories [28]. Our VAS depicted a 100-point vertical thermometer ranging from 0: 'worst imaginable health state' (lower anchor) to 100: 'best imaginable health state' (upper anchor), the standard EuroQoL-format [29]. Each respondent was asked to draw a horizontal line on the VAS to indicate where the combined maternal and neonatal health state vignette should be positioned, taking the top and bottom anchors into consideration.

The aim of the TTO method is to elicit the maximum amount of time in full health that respondents are willing to trade to avoid a suboptimal health state [30]. Our TTO method involved a two-step procedure: first, the respondent had to state how much maternal time he/she was roughly willing to give in and, second, given the rough indication, how much maternal time he/she was exactly willing to trade (see Additional file 3) [31]. We specifically asked respondents to state how much time of the mother's life in full health he/she was maximally willing to trade off in order to attain full health for both mother and infant, given their health states as presented in the vignette. Respondents could trade-off between 0 days and 10 years of the mother's life.

The aim of DCE is to derive patients' preferences for a number of different aspects ('attributes') of a health state by presenting hypothetical choices between two or more scenarios in which the levels of the attributes are systematically varied [32]. In our study, respondents were invited to choose the best one of two alternative vignettes (forced choice) within a vignette pair. For an example of one vignette, see Additional files 1 and 2.

### Study procedures

The study consisted of group sessions with 6 to 16 participants per group. There were two sessions with laypersons, two sessions with patients, and three sessions with professionals. The participants within each session were of the same respondent group. Each session was conducted by a trained moderator (DB, GJB, JAH, MFJ) who followed a detailed protocol adapted from the Dutch Disability Weights [33], MiDAS [27] and IBIS [34] protocols. Ethical consideration was not deemed necessary for this type of study.

In the group sessions, participants were invited to value the first 20 vignettes (18 single vignettes (9 paired vignettes for DCE), plus the best and worst possible vignettes) with first DCE, then VAS, and finally TTO. We explained the vignettes thoroughly, and respondents could practice on some sample vignettes in order to get used to the layout vignettes, the meanings of the used colours, and the weighting of the health states. The DCE task took about 10 minutes, the VAS task 15 minutes and the TTO task about 20 minutes. After the valuation tasks, participants filled out a questionnaire on background characteristics and their obstetric history.

Each session was followed by an individual home assignment. In the individual home assignment, the participants valued the remaining 26 vignettes: 22 single vignettes (11 paired vignettes for DCE), 4 single retest vignettes for VAS and TTO and 5 paired retest vignettes for DCE. They valued the vignettes in the same order as in the group session (first DCE, then VAS, and finally TTO). Finally, they completed a questionnaire on the user-feasibility of the written and visual components of the vignettes (response mode: 'comprehensible', 'neutral', 'incomprehensible'); the reference handout; comprehensibility of the five individual attributes; difficulty of each valuation method (response mode: 'easy', 'not easy but not difficult', 'difficult'); and the self-reported amount time needed to complete the home assignment. A telephone number was provided which the participants could dial if they needed assistance with the tasks or the questionnaires.

### Analysis

We measured feasibility, reliability, and comparability of each combination of group and valuation method.

Regarding feasibility, differences between groups of the time needed to complete the home assignment was calculated using one-way analysis of variance (ANOVA) followed by Tukey's post hoc test. Linear regression analysis was used to determine the impact of sex, age, educational level, and respondent group on the time needed to complete the home assignment. Feasibility ratings between groups were compared with the χ2 test or Fisher's Exact Test.

Reliability was investigated by generalizability theory (G-theory) with restricted maximum likelihood estimation (REML) was used to determine the variation explained by respondent group in the VAS and TTO valuations. The test-retest reliabilities of the TTO and VAS were analyzed per group using intra-class correlation coefficient (ICC; two-way random effects, single measures, absolute agreement; 95% CI). The DCE test-retest reliability was assessed using Cohen's unweighted kappa (κ). Within-group consistencies of the VAS and TTO were calculated per group using ICC (two-way random effects, single measures, absolute agreement; 95% CI) to measure the rate of consensus within each group.

To measure comparability, crude VAS (vas) and TTO (tto) scores were conventionally transformed into a 0-1 score as follows, where 1 represents the optimum [35]:

(1) VAS = (vas/100)

(2) TTO = 1-((tto/3650)^(1.61))

(3) DCE = Σ βXi

The DCE score for a health state was indirectly derived by adding up all attribute level coefficients of the health state (βXi is the coefficient of attribute X, level i). The mean transformed (VAS and TTO) and the indirectly derived (DCE) vignette scores were calculated for all 46 presented vignettes. The correlation between each two valuation methods per respondent group was plotted to visualize group clustering in valuations and to expose valuation tendencies per group-method combination.

The relative attribute weights (coefficients) were calculated per group for the VAS and TTO using linear regression of the transformed VAS and TTO scores, and by application of multinomial logit (conjoint analysis) on the DCE scores. ICCs were interpreted according to the guidelines of Landis and Koch [36]. The estimated relative attribute weights were compared between groups within methods, and between methods within groups, with Kendall's Tau-b correlation coefficient.

Analyses were conducted using SPSS 15.0 for Windows (SPSS Inc). Multinomial logit was performed using SAS 9.1.2 (SAS Institute Inc). A p-value < 0.05 (two sided) was considered to indicate statistical significance.

## Results

### Baseline characteristics

Participant's characteristics are shown in Table 1. Patients (mean age: 32 years, range 26-39 years) gave birth between 16 and 3 months prior to study participation. The group of obstetrical care professionals (mean age: 33 years, range 20-60 years) consisted of 11 (37%) gynaecologists, 5 (17%) midwives, and 14 (47%) residents in gynaecology. The mean number of years of medical obstetric experience was 8 (range 0.4-28.0). The group of laypersons consisted of 10 men and 17 women (mean age 55 years, range 22 to 78 years). There was enough variation in socio-economic status within the groups of laypersons and patients.

**Table 1:** Baseline characteristics of the participating patients, obstetrical care professionals (gynecologists, midwives, gynecological residents) and laypersons; N = 81.

	Patients *n = 24*	Professionals *n = 30*	Laypersons *n = 27*
Mean age (SD)	32.0 (3.8)	33.2 (10.4)	54.6 (16.6)
Female (%)	24 (100.0)	21 (60.0)	17 (63.0)
At least one child (%)	24 (100.0)	-	22 (81.5)
**Obstetric history:**			
- GH or PE * (%)	15 (62.5)	-	6 (22.2)
- IUGR ** (%)	14 (58.3)	-	1 (3.7)
- Complications during delivery (%)	23 (95.8)	-	14 (51.9)
- Maternal complications post partum (%)	14 (58.3)	-	11 (40.7)
- Neonatal complications postpartum (%)	12 (50.0)	-	7 (25.9)

* GH = Gestational hypertension; PE = Pre-eclampsia

** IUGR = Intrauterine growth restriction

### User-feasibility

The response rate to the home assignment was 96% for the patients, 73% for the obstetrical care professionals (residents: n = 12, 86%; gynaecologists: n = 7, 64%; midwives: n = 3, 60%) and 89% for the laypersons.

The mean amount of time needed to complete the home assignment was 45 minutes for the patients, 49 minutes for the obstetrical care professionals, and 75 minutes for the laypersons (laypersons versus patients p = 0.001; laypersons versus obstetrical care professionals, p = 0.007). Respondent's age had a significant impact on the amount of time (in minutes) needed to complete the home assignment (β = 0.315; p = 0.034, for higher age) while sex and education did not (females: β = 0.065; p = 0.598; > 11 years of education: β = 0.182; p = 0.168).

Comprehensibility of the vignettes was equal across groups (p = 0.861) and the visual and the written components of the vignettes were equally comprehensible across groups (p = 0.549). The DCE was overall rated as 'easy', the VAS as 'not easy but not difficult', and the TTO as 'difficult' (no differences between groups; p = 0.611, p = 0.746 and p = 0.738, respectively).

### Reliability

G-theory variance components for the VAS and TTO scores are shown in Table 2. The sum of variance explained by health state attributes was 66.0% (VAS) and 62.3% (TTO). 'Neonatal health postpartum' explained the highest proportion of variance (VAS: 53.4%; TTO: 61.7%). The proportion of variance explained by respondent group was 15.0% (VAS) and 19.6% (TTO), including 1.2% (VAS) and 14.2% (TTO) interaction effects of respondent group with maternal and neonatal outcome attributes.

**Table 2:** Estimated variance components in percentages of the respondent group, respondent characteristics and health state attributes for the transformed VAS and TTO

*Source of variation*	*VAS (%)*	*TTO (%)*
Respondent group (G)	5.61	0.65
Respondent * G	8.14	4.77
Respondent age	1.70	0.39
Respondent sex	1.33	1.17
Maternal health ante partum (A1)	0.23	0.10
Time between diagnosis and delivery (A2)	0.22	0.00
Process of delivery (A3)	1.98	0.23
Maternal outcome (A4)	9.46	0.31
Neonatal outcome (A5)	52.87	47.52
A4 * G	0.65	0.03
A5 * G	0.57	14.14
Residual (*e*)	17.23	32.85

Table 3 shows the test-retest and the within-group reliability. Time between test and retest ranged from 3 to 16 days. Overall test-retest reliability coefficients indicated substantial agreement for the VAS, with small variance between groups. Overall test-retest reliability for the TTO was moderate, with large variance between groups; the test-retest reliability was substantial for patients, moderate for laypersons and low for obstetrical care professionals. The DCE had low to very low test-retest reliabilities, overall as well as across groups.

**Table 3:** Test-retest reliability and within-group reliability intra class correlation coefficients (ICCs) for the VAS and TTO and Cohen's kappa (κ) for the DCE by respondent group and valuation method.

	Patients	Professionals	Laypersons	Overall	Range
**Test-retest reliability**					
VAS	0.73	0.61	0.62	0.69	0.11
TTO	0.74	0.24	0.51	0.46	0.50
DCE	0.18	0.15	0.37	0.25	0.22

**Within-group reliability**					
VAS	0.70	0.70	0.73	0.66	0.03
TTO	0.20	0.60	0.33	0.25	0.40
DCE	0.60	0.56	0.76	0.62	0.20

VAS = visual analogue scale; TTO = time trade-off; DCE = discrete choice experiment.

The overall within-group reliability coefficients indicated substantial agreement on the VAS; all groups displayed substantial within-group reliability. Overall within-group reliability was low for the TTO, with large differences between obstetrical care professionals versus patients and laypersons. The DCE had overall substantial within-group reliability, with some variation between groups.

### Comparability

**Figure 1:**
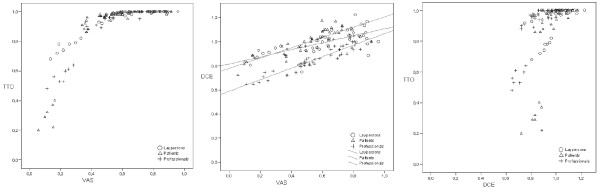
**A-C: Scatter plots of valuation scores of 46 vignettes per group of the TTO and VAS (1A), indirectly derived DCE and VAS with linear fit lines per group (1B), and transformed TTO and indirectly derived DCE (1C)**.

Figures 1A, B and 1C display the valuations of each group, for each pair of valuation methods respectively. The association between the transformed VAS and TTO scores per group is shown in Figure 1A. Participants are apparently less willing to trade off maternal life time for health states with VAS score > 0.50, a pattern consistent across respondent groups. For health states with VAS score < 0.50, all groups are willing to trade off maternal life time, but to a various degree; patients are generally willing to trade off more time than laypersons. The transformation of the TTO scores using formula (2) did not result in the intended linearity of the scores.

Figure 1B depicts the association between VAS and DCE scores per group. Over the entire VAS spectrum, patients and laypersons express systematically higher preference scores (in terms of DCE) than the obstetrical care professionals. The linear regression lines of the patients and obstetrical care professionals are parallel, with patients displaying a systematic higher valuation. The scores of laypersons are more in agreement with those of the patients at the lower end of the VAS, but more in agreement with the obstetrical care professionals at the upper end of the VAS spectrum (VAS > 0.75).

The scale variance heterogeneity was tested for VAS using the anchoring (both best and worst) health state vignettes. The worst and best possible vignettes resulted respectively in mean VAS scores of 0.13 and 0.96 for lay people, 0.05 and 0.92 for patients, and 0.11 and 0.91 for professionals. Using ANOVA with Tukey's post hoc test, the worst vignette differed between lay people and patients (p = 0.029) and the best vignette differed between lay people and patients (p = 0.026) and between lay people and professionals (p = 0.007).

Figure 1C shows the relationship between the transformed TTO and DCE valuations. For DCE scores > 0.90, respondents are less willing to trade off maternal time on the TTO; this pattern is present in all groups. For DCE scores < 0.90, all groups are willing to trade off maternal life time. Patients are again relatively more willing to trade off maternal life time than laypersons and professionals. From these figures it is apparent that the threshold score to trade off depends on the method chosen.

**Figure 2:**
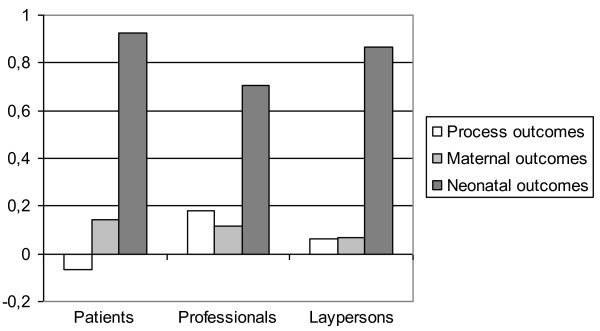
**Aggregated relative disability weight per attribute category (process, maternal, and neonatal outcome), per respondent group**.

Table 4 presents the relative attribute weights (coefficients) for each valuation method per respondent group. Increasing relative weights with decreasing optimality of the health states is overall most consistent in the VAS. The TTO suggests a skewed relationship between health state and relative weight; while mild and moderate health states have low relative weights, severe health states (neonatal outcome) have very high relative weights; an observation that is consistent in all groups. The DCE shows another pattern: relative weights increase with the severity of the health states (neonatal outcome), but less strongly than VAS and TTO. Figure 2 shows the aggregated relative weights (i.e. mean relative weight over the methods) per respondent group, per attribute category: process, maternal outcome, and neonatal outcome.

**Table 4:** VAS, TTO, and DCE relative attribute level weights (coefficients) per respondent group (N = 81)

Attribute and level (baseline)	VAS			TTO			DCE		
	Patients	Professionals	Laypersons	Patients	Professionals	Laypersons	Patients	Professionals	Laypersons
**Maternal health ante partum **(normal)									
- Moderate	-0.020	-0.010	-0.006	0.025	0.023	0.003	0.003	0.013	0.031
**Time between diagnosis and delivery **(3 days)									
- 1 week	-0.024**	-0.016*	-0.003	0.005	-0.003	0.017	0.053*	0.050	0.054*
- 2 weeks	-0.013	-0.022*	-0.014	-0.003	-0.020	-0.022	-0.042	0.001	-0.057*
**Process of delivery **(Cervical)									
- Induction, cervical	-0.081**	-0.069**	0.045*	0.028	0.044	0.080	0.023	-0.110	0.176
- Vacuum	-0.023*	-0.005	0.004	0.009	0.011	0.019	0.039	-0.065	-0.058
- Induction, vacuum	-0.023	0.008	-0.006	0.025	0.035	0.030	0.113	-0.065	0.008
- Cesarean section (planned)	-0.045**	-0.005*	-0.047*	0.025	0.021	0.008	0.093	-0.131	-0.066
- Cesarean section (not planned)	-0.042**	-0.069*	-0.047*	-0.019	0.006	-0.006	0.093	-0.076	-0.081
- Induction, Cesarean section (not planned)	-0.042**	-0.045	-0.047*	-0.030	-0.030	-0.047	0.025	-0.078	-0.089
**Maternal outcome **(3 days moderate)									
- 3 days severe and 4 days moderate	-0.033**	-0.034**	-0.030*	-0.010	-0.008	0.014	-0.072	-0.021	0.053
- 7 days severe and after 1 year moderate	-0.070**	-0.146**	-0.113*	-0.024	-0.066	-0.052	-0.126*	-0.116	-0.005
**Neonatal outcome **(No complications)									
- 7 days moderate	-0.042**	-0.031**	-0.022*	0.008	-0.003	-0.003	0.076**	-0.016	0.079**
- 3 days severe and 7 days moderate	-0.087**	-0.077**	-0.072**	0.001	-0.015	0.003	0.010	-0.001	0.061
- 10 days severe and after 1 year moderate	-0.169**	-0.154**	-0.179**	-0.099**	-0.101**	-0.074	-0.093**	-0.087	-0.108**
- 3 days severe and death	-0.287**	-0.309**	-0.366**	-0.688**	-0.615**	-0.623**	-0.138**	-0.172*	-0.074**

* p ≤ .05 ** p ≤ .01

Kendall's Tau-b correlations of the relative attribute-level weights between respondent groups and between valuation methods are shown in Table 5. The correlations of relative weights between groups were highest for VAS and lowest for DCE. The relative weights elicited with VAS and TTO have highest comparability between obstetrical care professionals and laypersons (VAS τ = 0.76; TTO τ = 0.61). The relative weights estimated with DCE consistently show lowest comparability, regardless of the pair of groups compared.

**Table 5:** Kendall's Tau-b correlations of the relative attribute weights between groups and between methods.

	Patients - Professionals	Patients - Laypersons	Professionals - Laypersons
**VAS**	0.64	0.69	0.76
**TTO**	0.59	0.46	0.61
**DCE**	0.41	0.12	0.26

	**VAS - TTO**	**VAS - DCE**	**TTO - DCE**
**Patients**	0.37	0.28	0.30
**Professionals**	0.37	0.24	0.44
**Laypersons**	0.28	0.28	0.14

VAS = visual analogue scale; TTO = time trade-off; DCE = discrete choice experiment.

From the group perspective, correlations between relative weights obtained with each pair of valuation methods were consistently low to moderate across groups of respondents. VAS-TTO correlation was highest among patients and professionals (τ = 0.37). VAS-DCE was highest among patients and laypersons (τ = 0.28), while TTO-DCE correlation was highest among professionals (τ = 0.44). When comparing each two valuation methods across groups, the laypersons give the most inconsistent valuations between methods.

## Discussion

We investigated whether three relevant groups (patients, obstetrical care professionals, and laypersons) agreed on the valuations of a set of realistic complex obstetrical outcomes, using three widely used valuation methods. Within the groups, there is consensus among obstetrical care professionals but not among patients nor among laypersons about the valuation of the health states. Between groups, we found significant differences in terms of absolute values, but not in the overall ranking of the health attributes. Valuation patterns differed also between the groups, which was especially apparent in the TTO method.

The respondents of all groups rated the user-feasibility of the valuation methods consistently in the same order: The DCE was considered as easy, the VAS was not easy but not difficult, and the TTO was difficult to use. Moreover, all groups were equally able to understand the complex vignettes.

There were considerable group differences in test-retest reliability on the TTO: the TTO test-retest reliability was high for patients, intermediate for laypersons and low for professionals. All groups scored (very) low on the DCE, which could be due to the selection of rather complex test-retest choice sets [36]. In our previous study, the DCE test-retest results in a large group of laypersons were very good using the same type of vignettes but less complex choice sets [24]. Also considering the DCE's good test-retest results in other studies, the low test-retest performance in this study should be interpreted against the degree of choice difficulty (e.g. [37]). Remarkably, the obstetrical care professionals were least reliable over time on all three valuation methods. Regarding TTO, this is probably due to the relatively narrow ranges of valuations assigned by this group; a small shift in valuations has larger impact on test-retest reliability compared to the other groups.

Within-group consensus was good on VAS and DCE for all groups, but there were group differences in TTO; consensus was low for patients and laypersons but high for obstetrical care professionals [36]. This high consensus among obstetrical care professionals may be the result of their daily involvement in clinical decision-making, the following of medical protocols, and the process of reaching clinical consensus for medical treatments. This indicates that medical decision-making among obstetrical care professionals requires smaller group sizes when interested in group opinion, as compared to patients and laypersons.

The absolute valuations differed markedly between groups. Professionals assigned relatively most disability weight to process outcomes, while patients assigned most disability weights to maternal and neonatal outcome compared to the other groups. These results are in line with the results by Vandenbussche et al. (1999), who found that professionals were clearly antipathetic to caesarean section while patients had no overriding preference for type of birth [38]. Laypersons in our study valued process outcome and maternal outcome equally (Figure 2) and used significantly different scale ranges. In a meta-analysis, Peeters and Stiggelbout (2010) showed differences between patients and laypersons in VAS and TTO valuations but not between patients and professionals [39]. In our study, professionals had the highest contrast to the patient group.

Explanations for health-state valuation differences between groups have been extendedly described by Ubel, Loewenstein & Jepson (2003) and Stiggelbout & De Vogel-Voogt (2008). These authors argue that valuation differences between groups could relate to differences in interpretation of the vignettes, fundamental differences in opinion between groups regardless of valuation method, or to differences in the use of valuation methods [5,19]. Using this framework, we reason that our valuation differences are due to differences in interpretation; patients tend to assign valuations in the light of their own personal experience with pregnancy and childbirth, which is absent or less profound among laypersons and professionals. Also, patients and laypersons may not simply value overall health but a full life in the particular health state ('wellbeing'). Professionals, however, may over-value the process outcomes compared to patients and laypersons due to fundamental differences in opinion, which originate in their daily professional involvement with these processes. Our results are less likely to be influenced by different interpretations of the health states since we invested considerable effort in explaining the vignettes following a protocol. However, we cannot conclude whether the different answering patterns between groups reflect different points of view or fundamentally different opinions between groups (see also Figures 1A-C).

Three study limitations need to be discussed. First, it is unclear if our results can be generalized taking in consideration that we used complex health states which are common in obstetrics but rare in other health care domains. Second, one may argue that sample size per group is too small for valid comparisons. We judge this in our study not to be the case when comparing the groups with the use of VAS. The VAS showed that with these group sizes even small but statistically significant relative weights can be obtained (Table 4). Moreover, in a related obstetric health-state valuation study much smaller group sizes already proved to be sufficient [38]. However, on the TTO and DCE, group sizes may be debatable because some weights that were significant on VAS were not so on TTO or DCE. The TTO and DCE might therefore require larger groups; this is also evident from the within-group reliability coefficients, which are lower for the TTO and DCE than for the VAS (Table 3). Lastly, we could not establish any ordering effects of the valuation methods while we used a fixed order in the tasks: first the supposedly simple DCE, then the VAS, and then the most difficult TTO.

The particular choice of both respondent group and method affects results. Selecting one group over the other and one method over the other may result in exaggerated or underestimated health benefits [5,8,40,41]. Gold et al. [42] recommend the use of the societal perspective (represented by laypersons) for societal decision making (e.g. economic evaluation), and the patient's perspective for guideline development and patient decision making, each using a trade-off based method (e.g. TTO, DCE) to yield valuations. When using TTO to support decision making, large valuation differences are to be expected between groups and selection of respondent group is critical. This is especially true when severe health states are to be valued or when health improvements from severe to mild states are at stake. When using DCE, the valuation gap between patients and professionals is about constant irrespective of health state severity. This implies that patients and professionals assign different DCE valuations but that the valuation differences of health improvements remain unaffected. In contrast, DCE valuations of laypersons are somewhat lower for the same health state than those of patients or professionals. Societal decision making based on laypersons' DCE valuations could therefore yield a higher burden or lower effectiveness of interventions than guidelines or decisions based on patients' or professionals' valuations.

We infer that societal decision making among a group of laypersons should use TTO or DCE; individual decision making with patients should use VAS or TTO. Obstetrical care professionals should not be asked to complete a TTO, due to a lack of consistency over time. Especially TTO has large power to discriminate between groups. For clinicians and policy makers it is important to understand that patients often make their decisions based on other values that clinicians do. This is especially in obstetric decisions, where women value their child's health as much more important as their own. Summarizing, in our context the effect of respondent group was substantial, but the effect of the valuation method remained dominant.

## Conclusions

• Decision-making in obstetrics is affected majorly by the research methodology, but to a lesser extent also by the respondent group.

• There were interaction effects between methodology and respondent group.

• Societal decision making among laypersons should use the Time Trade-Off (TTO) or Discrete Choice Experiment (DCE).

• Individual patients should use Visual Analogue Scale (VAS) or TTO.

## List of Abbreviations

TTO: Time Trade-Off; VAS: Visual Analogue Scale; DCE: Discrete Choice Experiment; GH: Gestational Hypertension; PE: Pre-eclampsia; IUGR: Intra-Uterine Growth Restriction.

## Competing interests

The authors declare that they have no competing interests.

## Authors' contributions

DB, EB and GJB participated in the design of the study. DB, EB, and BWJM participated in the coordination of the data collection and data analysis. DB, EB, GJB, and BWJM read and approved the final manuscript.

## Pre-publication history

The pre-publication history for this paper can be accessed here:

http://www.biomedcentral.com/1471-2393/11/93/prepub

## Supplementary Material

Additional file 1 **Example of a vignette**. An example of one of the health state vignettes that has been presented to the participants. Click here for file

Additional file 2 **Reference handout**. The reference handout which has been handed out to the participants explaining the meaning of the figures and colours of the vignettes. Click here for file

Additional file 3 **Example of a 10-year time trade-off (TTO)**. The 10-year time trade-off (TTO) we used involving a two-step method: first the participants stated how much time they were roughly willing to trade-off, and then they stated how much time they were exactly willing to trade-off for each of the vignettes. Click here for file
