# Supplementary figures and images for: Obstetrical outcome valuations by patients, professionals, and laypersons: differences within and between groups using three valuation methods
Source: BMC Pregnancy Childbirth. 2011 Nov 12;11:93. doi: 10.1186/1471-2393-11-93 (PMC3226638; doi:10.1186/1471-2393-11-93)

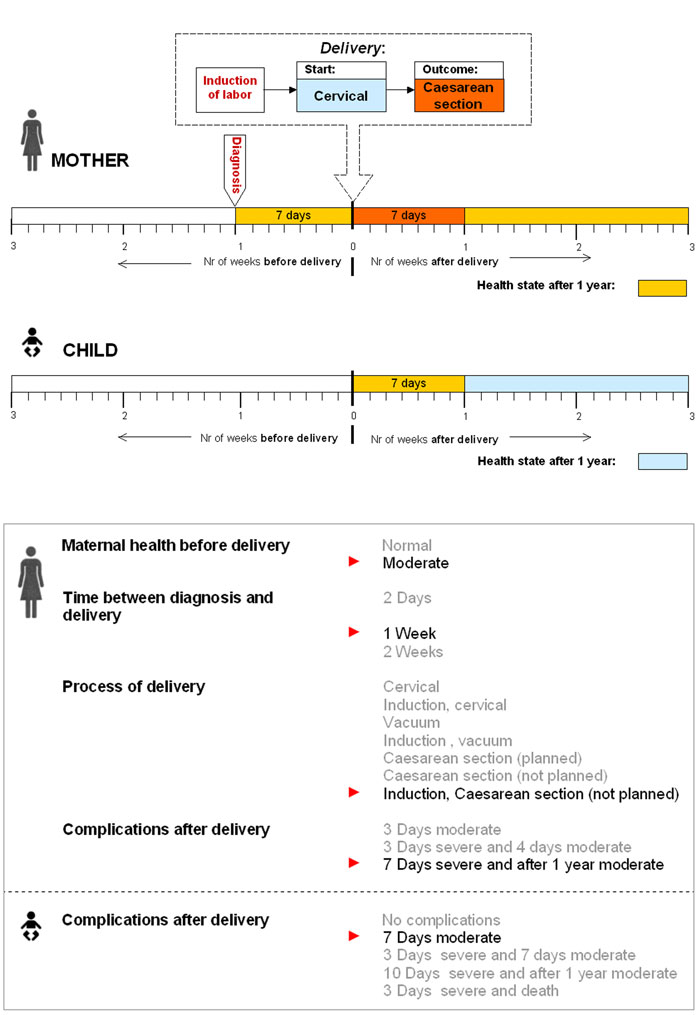

Supplement: Additional file 1 — Example of a vignette. An example of one of the health state vignettes that has been presented to the participants. [file 1471-2393-11-93-S1.DOC]

*
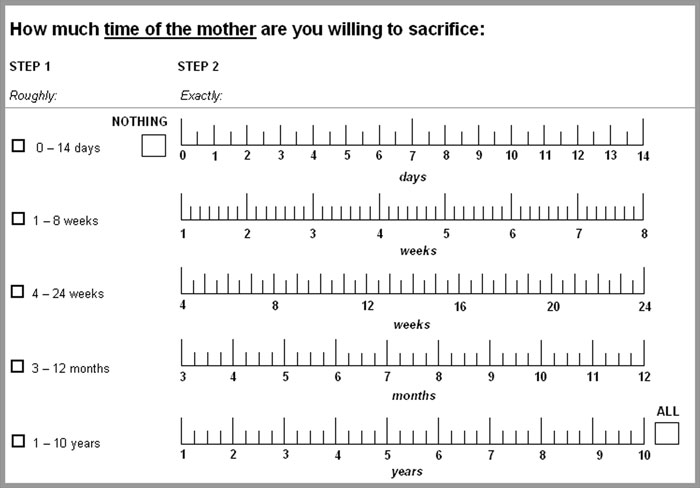
*

Supplement: Additional file 3 — Example of a 10-year time trade-off (TTO). The 10-year time trade-off (TTO) we used involving a two-step method: first the participants stated how much time they were roughly willing to trade-off, and then they stated how much time they were exactly willing to trade-off for each of the vignettes. [file 1471-2393-11-93-S3.DOC]
