# Supplementary material for: Obstetrical outcome valuations by patients, professionals, and laypersons: differences within and between groups using three valuation methods
Source: BMC Pregnancy Childbirth. 2011 Nov 12;11:93. doi: 10.1186/1471-2393-11-93 (PMC3226638; doi:10.1186/1471-2393-11-93)
Supplement: Additional file 2 — Reference handout. The reference handout which has been handed out to the participants explaining the meaning of the figures and colours of the vignettes. [file 1471-2393-11-93-S2.DOC]

|  | ***Written component*** | ***Visual component*** | ***Explanation*** |
| --- | --- | --- | --- |
| **Diagnosis** |  | diagnosis | The doctor confirms a deterioration of the physical health of the mother or the unborn child. At the moment there is no medical necessity to take action, there is a risk of further deterioration. |
| **Physical condition before delivery** | Normal |  | Normal physical condition during pregnancy. |
|  | Moderate |  | Prescribed bed rest by the doctor / moderate anxiety / moderate stress / some physical restraints |
| **Risk period** | Number of days | … days | Number of days in which the mother has the risk of deterioration of the health state and in which there is medical monitoring. |
| **Process of delivery** | Cervical | **Start:**  **Vaginaal**  **Cervical**  **Spontaneous**  **Result:**  **Vaginaal**  **Cervical** | Natural delivery without assisting instruments or operation. |
|  | Induction, Cervical | **Start:**  **Vaginaal**  **Cervical**  **Intravenous induction**  **Result:**  **Vaginaal**  **Cervical** | The uterus is stimulated with hormones, which sometimes gives strong and painful contractions. The result is a Cervical delivery. |
|  | Vacuum | **Start:**  **Vaginaal**  **Cervical**  **Result:**  **Vaginaal**  **Vacuum**  **Spontaneous** | A Cervical delivery with instrument assistance of a vacuum pump. |
|  | Induction, vacuum | **Start:**  **Vaginaal**  **Cervical**  **Result:**  **Vaginaal**  **Vacuum**  **Intravenous induction** | The uterus is stimulated with hormones, which sometimes gives strong and painful contractions. The result is a Cervical delivery with instrument assistance of a vacuum pump. |
|  | Caesarean section (planned) | **Start:**  **Vaginaal**  **Caesarean section**  **Result:**  **Vaginaal**  **Caesarean section** | The doctor elects an operative intervention because of the worsening of the situation, sometimes urgent, with local anaesthesia. There is a 50% chance to have a Caesarean section in a subsequent pregnancy. |
|  | Caesarean section (not planned) | **Start:**  **:**  **Vaginaal**  **Cervical**  **Result:**  **Vaginaal**  **Caesarean section**  **Spontaneous** | Operative intervention because the Cervical delivery stagnates, always urgent, and with complete anaesthesia. There is a 50% chance to have a Caesarean section in a subsequent pregnancy. |
|  | Induction, Caesarean section (not planned) | **Start:**  **Vaginaal**  **Cervical**  **Result:**  **Vaginaal**  **Caesarean section**  **Intravenous induction** | The uterus is stimulated with hormones, which sometimes gives strong and painful contractions. Then there is an operative intervention because the Cervical delivery stagnates, always urgent, and with complete anaesthesia. There is a 50% chance to have a Caesarean section in a subsequent pregnancy. |
| **Complications after delivery (both mother and child)** | No complications |  | No inconvenience or complications / normal health state |
|  | Moderate complications |  | Recovery period / lingering symptom / moderate pain / moderate complications |
|  | Severe complications |  | Severe pain / severe complications / hospital admission is necessary |
